# *Gardnerella vaginalis* in Recurrent Urinary Tract Infection Is Associated with Dysbiosis of the Bladder Microbiome

**DOI:** 10.3390/jcm11092295

**Published:** 2022-04-20

**Authors:** Jeong-Ju Yoo, Ju Sun Song, Woong Bin Kim, Jina Yun, Hee Bong Shin, Mi-Ae Jang, Chang Beom Ryu, Sung Shin Kim, Jun Chul Chung, Jung Cheol Kuk, Eung Jin Shin, Ho-Yeon Song, Byung Chul Yu, Eek-Sung Lee, Seongho Ryu, Jae Heon Kim, Sung Soo Jung, Young Ho Kim, on behalf of the SMS (Soonchunhyang Microbiome Multi-Disciplinary Study Group)

**Affiliations:** 1 Department of Internal Medicine, Soonchunhyang University Bucheon Hospital, Bucheon 14584, Korea; puby17@naver.com (J.-J.Y.); 19983233@schmc.ac.kr (J.Y.); ryuchb@schmc.ac.kr (C.B.R.); nephroybc@schmc.ac.kr (B.C.Y.); sung-soo.jung@schmc.ac.kr (S.S.J.); 2 GC Genome, Department of Laboratory Medicine, Green Cross Laboratories, Seoul 16924, Korea; jssong@gcgenome.com; 3 Department of Urology, Soonchunhyang University Bucheon Hospital, Bucheon 14584, Korea; woongbins@schmc.ac.kr; 4 Department of Laboratory Medicine and Genetics, Soonchunhyang University Bucheon Hospital, Bucheon 14584, Korea; shinhb@schmc.ac.kr (H.B.S.); miaeyaho@schmc.ac.kr (M.-A.J.); 5 Department of Pediatrics, Soonchunhyang University Bucheon Hospital, Bucheon 14584, Korea; kimss@schmc.ac.kr; 6 Department of Surgery, Soonchunhyang University Bucheon Hospital, Bucheon 14584, Korea; capcjc@schmc.ac.kr (J.C.C.); kjc1110@schmc.ac.kr (J.C.K.); colon@schmc.ac.kr (E.J.S.); 7 Department of Microbiology and Immunology, School of Medicine, Soonchunhyang University, Cheonan 31151, Korea; songmic@schmc.ac.kr; 8 Department of Neurology, Soonchunhyang University Bucheon Hospital, Bucheon 14584, Korea; eeksung@schmc.ac.kr; 9 Department of Integrated Biomedical Science, School of Medicine, Soonchunhyang University, Cheonan 31151, Korea; ryu@sch.ac.kr; 10 Department of Urology, Soonchunhyang University Seoul Hospital, Seoul 04401, Korea; piacekjh@schmc.ac.kr

**Keywords:** *Gardnerella*, 16S rRNA next-generation sequencing, urinary tract infection, microbiome, recurrent cystitis, bladder

## Abstract

Recent studies on the urine microbiome have highlighted the importance of the gut–vagina–bladder axis in recurrent urinary tract infection (rUTI). In particular, the role of *Gardnerella* as a covert pathogen that activates *E. coli* in animal experiments has been reported. Herein, we conducted a human bladder microbiome study to investigate the effect of *Gardnerella* on rUTI. Urine 16S ribosomal RNA gene sequencing via transurethral catheterization was conducted in the normal control group (NC) (*n* = 18) and rUTI group (*n* = 78). The positive detection rate of *Gardnerella* species did not differ between the NC and rUTI groups (22.2% vs. 18.0%, *p* = 0.677). In addition, the *Gardnerella*-positive NC and *Gardnerella*-positive rUTI groups showed similar levels of microbiome diversity. The *Gardnerella*-positive group was categorized into three subgroups: the *Escherichia*-dominant group, *Gardnerella*-dominant group, and *Lactobacillus*-dominant group. All of the *Escherichia*-dominant groups were associated with rUTI. The *Gardnerella*-dominant or *Lactobacillus*-dominant groups expressed rUTI with symptoms when risk factors such as the degree of *Gardnerella* proliferation or causative agents of bacterial vaginosis were present. The presence of *Gardnerella* in the urine is considered to be related to rUTI depending on other risk factors. New guideline recommendations regarding antibiotic selection based on a novel method to detect the cause of rUTI may be required to reduce antibiotic resistance.

## 1. Introduction

Urinary tract infections (UTI) are approximately eight times more common in women than in men [1], and 50–60% of adult women will experience at least one UTI in their lifetime [2,3]. Of these, 30% of UTI patients develop recurrent urinary tract infection (rUTI) within 6 months [2,3]. Clinically, UTI can develop into systemic diseases such as severe kidney disease (acute kidney injury, kidney atrophy, end-stage renal disease) and sepsis. Moreover, UTI is problematic as it can affect quality of life or cause depression [4,5,6]. UTI treatment is based on the assumption that urine is sterile, and antibiotics are the treatment of choice based on standard urine culture. However, in reality, the causative bacteria of many patients may not be detected by standard urine culture methods, and rUTI develops in approximately 25–30% of patients despite the use of antibiotics [7].

Traditionally, colonization of the vaginal introitus or periurethra by pathogenic gut bacteria is known to cause rUTI by retrograde infection [8,9]. Due to this gut–bladder axis etiology, the vaginal microbiome, particularly *Gardnerella vaginalis*, is frequently excluded as a causative agent of UTIs, although they can also cause the infection. The clinical significance of *G. vaginalis* has been underestimated to date, due to its low detection rate in traditional urine culture [10]. In general, uropathogenic *Escherichia coli* (UPEC) is known as the most common causative agent of rUTI, and thus clinical studies on polyinfection by Gram-positive bacteria or co-infective UTI by vaginal microbiota are few in number [11,12].

Recently, 16S ribosomal RNA gene amplification and sequencing and the enhanced quantitative urine culture (EQUC) technique have revealed that a complex bladder microbiome exists in addition to classical bacteria [13]. Through a previous pilot study, we demonstrated the difference in bacterial diversity and patterns between rUTI and acute uncomplicated cystitis [14]. Due to these new techniques, a new disease pathway through gut–vagina–bladder crosstalk has gained focus, apart from the traditional mechanism of UTI, which was mainly caused by gut–bladder crosstalk [8,9,15,16]. Such changes in study perspectives can provide good therapeutic clues, particularly in rUTI, which is difficult to treat with frequent recurrence.

Recent animal studies have shown that *Gardnerella*, a major pathogen of bacterial vaginosis, triggers UPEC and causes rUTI [15,17]. Based on these results, we conducted a 16S ribosomal RNA gene sequencing study to investigate the differences in the urinary microbiome of rUTI and the effect of *Gardnerella*, a major strain of bacterial vaginalis, in patients with rUTI.

## 2. Materials and Methods

### 2.1. Patients and Study Protocol

Between April 2020 and June 2021, we collected information on patients who underwent the urine next-generation sequencing (NGS) test, 16S ribosomal RNA gene amplification. Patients who fulfilled the following inclusion criteria were eligible for this study: (a) patients 20 years of age or older, (b) who underwent urinalysis, urine culture, and urine NGS. Patients with single acute uncomplicated cystitis, anatomical or structural abnormalities such as a prolonged indwelling catheter, pregnancy, or urinary stone were excluded from the study. In addition, patients with intrauterine contraceptive devices, vaginitis, and vaginal discharge were excluded. To prevent the study results from being affected by changes in the microbiological environment in the bladder due to antibiotics, patients who were prescribed antibiotics for recurrent UTI or who had taken them within the last 4 weeks were excluded from this study. As a result, 96 patients who met the criteria were included in the study.

The patients were divided into two groups according to the following definitions. The normal group consisted of patients displaying no cystitis symptoms for at least the past year, and those that underwent a urine NSG test for the purpose of examination at a health promotion center, with no abnormal findings in abdominal image or urinalysis. The rUTI group was defined as consecutive patients who visited the outpatient clinic with symptomatic rUTI between April 2020 and June 2021. rUTI was defined as positive repetitive urine cultures (twice in six months, or three times in a year) with typical cystitis symptoms (low abdominal discomfort or pain, dysuria, frequency, urgency, hematuria, postvoid sense of residual urine). The previous urine culture results of rUTI patients were verified as medical records, and the urine culture positive rate was 52%. The most common causative bacteria were *E. coli* 64%, Klebsiella 10%, Enterococcus 8%, S. aureus 4%, Streptococcus 3%, and others, such as Proteus. However, in 48% of rUTI cases, the causative organism was not identified in urine culture even though the patient complained of urinary symptoms. Therefore, we found that it is difficult to diagnose the cause of rUTI patients using traditional culture methods only. This suggests that strains other than the traditionally known strains may affect rUTI. Therefore, in a total of 78 patients (30 patients with 2 or more occurrences in 6 months and 48 patients with 3 or more occurrences within 1 year), NGS sequencing and urine culture were performed simultaneously at the time of the next UTI recurrence.

The study protocol was approved by the Institutional Review Board of Soonchunhyang University Bucheon Hospital (IRB number SCHBC 2021-10-011-01). The study protocol conformed to the ethical guidelines of the World Medical Association Declaration of Helsinki. Informed consent was waived from the IRB of Soonchunhyang University Bucheon Hospital due to the retrospective design of this study.

### 2.2. DNA Extraction and 16S rDNA Sequencing

The overall process is similar to the previous research protocol [14]. All urine samples were collected by transurethral catheters. Upon collection, samples were immediately sent to the lab to be stored and refrigerated with boric acid, and transported to the genetic lab within one day. The urine specimens (25 mL) were centrifuged at 3300× *g* rpm for 30 min, and the urinary pellets were harvested and processed for DNA extraction using the MagMAX™ Microbiome Ultra Nucleic Acid Isolation Kit (ThermoFisher Scientific, Waltham, MA, USA) according to the manufacturer’s instructions. To check the contamination of the DNA extraction process, we performed a negative DNA extraction consisting of only reagents without urine specimens during the test setup.

Prepared DNA was used for 16S library construction using the NEXTflex 16S V4 Amplicon-Seq (Bioo Scientific, Austin, TX, USA), according to the manufacturer’s protocol. In detail, the amplification cycle was 8 cycles for PCR I amplification and 22 cycles for PCR II amplification, and each PCR clean-up used AMPure XP beads to purify the 16S V4 amplicon assay from free primers and primer dimer species. Final library products were quantified using Agilent D1000 screentape (Agilent Technologies, Santa Clara, CA, USA) and diluted to 4nM. Diluted library were pooled together and sequenced with the Miseq system (Illumina) using a paired-end 500-cycle kit.

### 2.3. Bioinformatics Analysis and Data Processing

We used QIIME 2 to analyze the 16S sequence data. Demultiplexed and primer-trimmed data were quality-filtered and denoised using DADA2 [18,19]. Amplicon sequence variants (ASVs) with fewer than 10 reads or present in only a single sample were removed, and taxonomy was assigned to each ASV using the naive Bayes machine learning taxonomy classifiers in the q2 feature classifier against the NCBI RefSeq database with taxonomic weight assembly using q2 clawback [20,21].

Contamination was removed separately for each pair of urine sample and negative control with the R package microDecon [22], which uses the proportions of contaminant operational taxonomic units (OTUs) or amplicon sequence variants (ASVs) in blank samples to systematically identify and remove contaminant reads from metabarcoding data sets. Although we applied decontamination bioinformatic pipelines, it was considered that the applying a relative abundance cut-off 1% was appropriate to more strictly identify the core microbiome as referring to other studies targeting low-biomass samples [23,24].

### 2.4. Statistical Analysis

The proportions of *Gardnerella* (+) urinary microbiota samples of the NC group and rUTI group were compared using the Chi-squared test. Next, we compared the characteristics of the microbial community between the *Gardnerella* (+) NC group and *Gardnerella* (+) rUTI group. Differences in alpha diversity between the *Gardnerella* (+) NC group and *Gardnerella* (+) rUTI group were analyzed based on Shannon’s diversity index using the Wilcoxon test. Principal coordinates analysis based on weighted Unifrac distances was used to construct a visualization of the data. Permutational multivariate analysis of variance (PERMANOVA) [23], implemented in the adonis function of the R/vegan package (v2.5–2, R version 4.1.0; The R Foundation for Statistical Computing, Vienna, Austria), was performed to identify the microbial community dissimilarity of the *Gardnerella* (+) NC group and *Gardnerella* (+) rUTI group. To discriminate *Gardnerella* (+) urine samples into subgroups according to microbial community similarity regardless of the disease group, we used the k-medoids clustering algorithm, which clusters samples with the smallest total pairwise distance [25]. The number of clusters was assessed with the silhouette method [26]. In addition, hierarchical clustering using complete linkage was performed to visualize relationships among the samples based on the similarity of microbial composition. The procedure was also operated on a phylogenetically informed distance matrix, which was computed using the weighted UniFrac metric. Through hierarchical clustering, a heatmap of the relative abundance of the compositional genera based on Spearman´s correlation coefficient was represented and each subgroup was named on the basis of the dominant member of the respective subgroup.

## 3. Results

### 3.1. Baseline Characteristics

The clinical characteristics and the urinalysis results of the patients are presented in Table 1. The patients were all female and the mean age was 54.5 ± 14.9 years. The NC group consisted of 18 patients, and the rUTI group was of 78 patients. The mean age of the rUTI group was slightly higher than that of the control group, but it was not statistically significant (56.2 vs. 47.1 years, *p* = 0.291). There were no differences between the groups in terms of menopause or diabetes.

### 3.2. Gardnerella Positive Detection Rate in the NC and rUTI Groups

Next, we compared the positive detection rate of *Gardnerella* in the NC and rUTI groups (Table 2). *Gardnerella* was detected in 18 of 96 patients (18.8%). Regarding the positive detection rate, there was no significant difference between the two groups, with 22.2% in the NC group and 18.0% in the rUTI group (*p* = 0.677).

### 3.3. Microbiome Diversity of the NC and rUTI Groups

**Figure 1:**
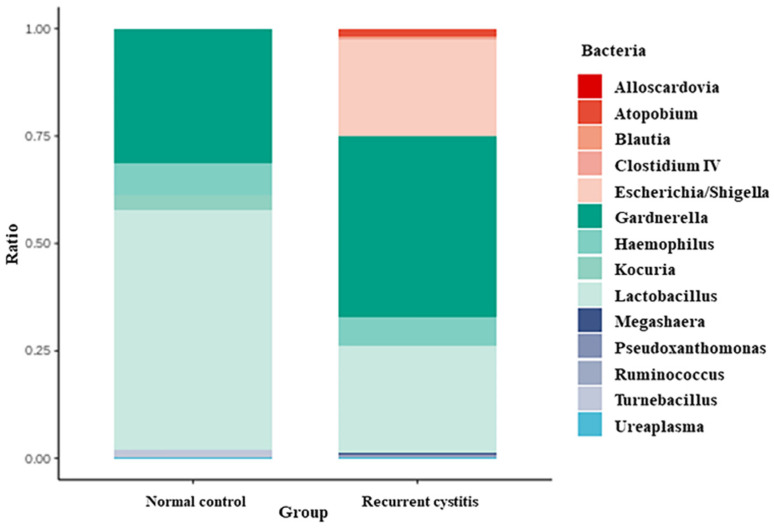
Relative abundance of urinary microbiota in *Gardnerella* (+) normal control group and *Gardnerella* (+) recurrent UTI group.

Bacteria frequently detected in *Gardnerella*-positive patients were classified according to the NC group and rUTI group, respectively (Table 3, Figure 1). In the *Gardnerella*-positive NC group, *Lactobacillus* (55.67%), *Gardnerella* (30.94%), *Haemophilus* (7.58%), and *Kocuria* (3.6%) groups were frequently detected. On the other hand, in the *Gardnerella*-positive rUTI group, *Gardnerella* (42.18%), *Lactobacillus* (24.87%), *Escherichia* (22.57%), and *Haemophilus* (6.52%) groups were frequently detected. In particular, *Atopobium*, *Megasphaera*, and *Ureaplasma*, known to be associated with bacterial vaginosis, were detected only in the rUTI group.

**Figure 2:**
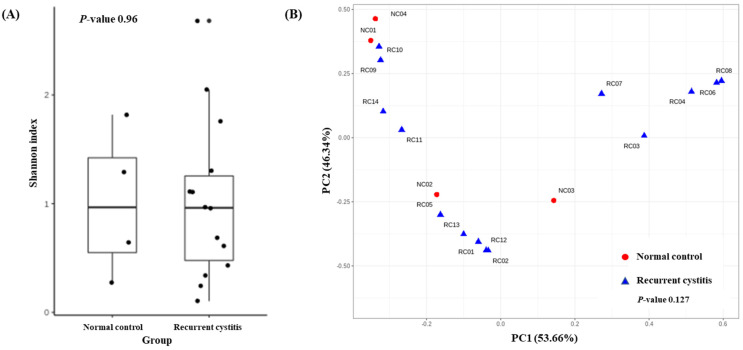
(**A**) Alpha diversity and (**B**) principal coordinate analysis based on weighted UniFrac distances in *Gardnerella* (+) normal control group and *Gardnerella* (+) recurrent UTI group.

To determine the distribution of various microorganisms present in one sample (alpha diversity), we calculated the Shannon index (Figure 2A). There was no significant difference regarding alpha diversity between the rUTI group and NC group (*p* = 0.96). To determine whether the microbial community was different between the two cystitis groups (beta diversity), we evaluated the weighted UniFrac distances (Figure 2B). The composition of the microbiome did not differ between the two groups (*p* = 0.127).

### 3.4. Three Urotypes of Bladder Microbiome Associated with Gardnerella

**Figure 3:**
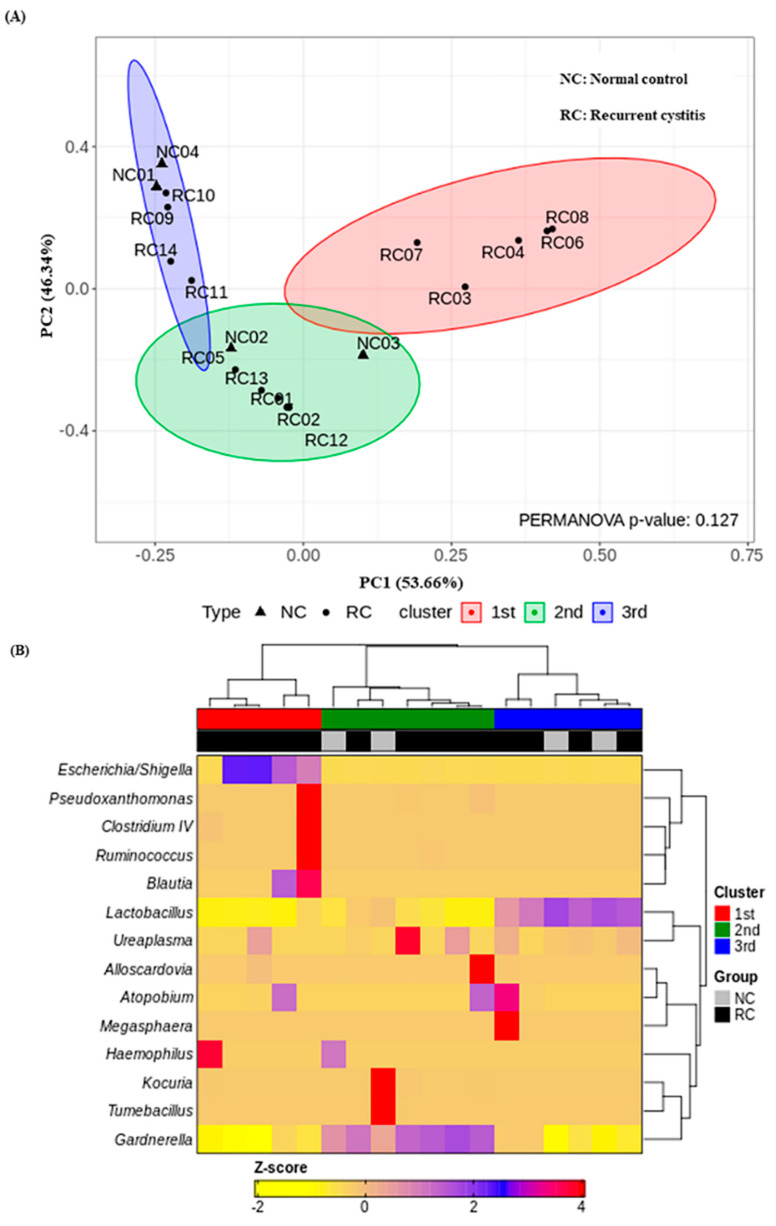

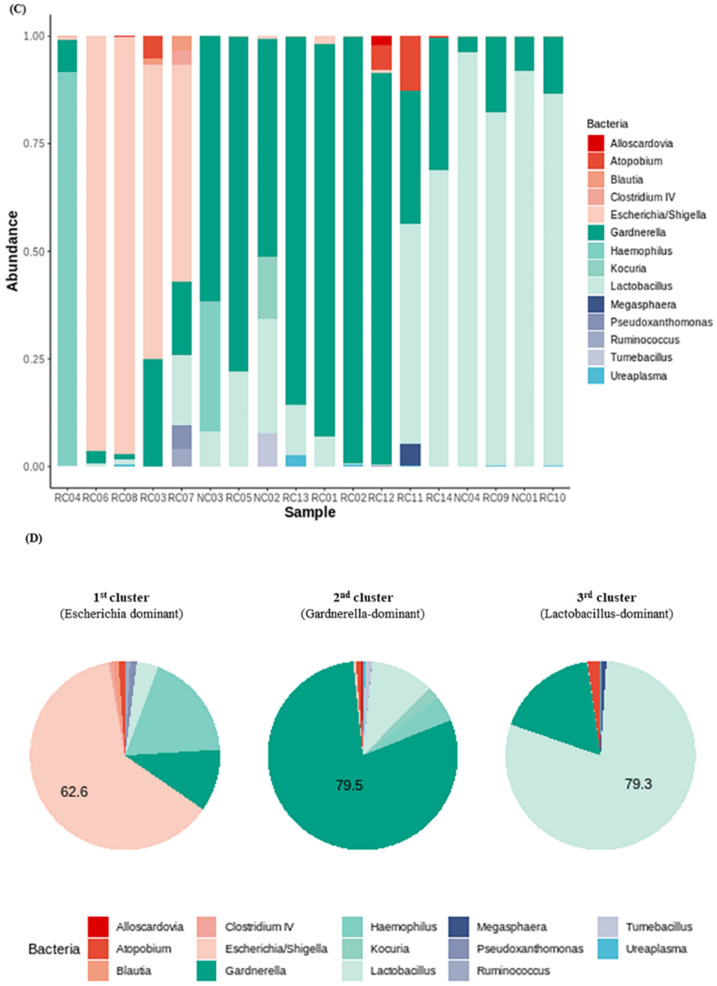
*Gardnerella* (+) urinary microbiota revealed three distinct subgroups by (**A**) K-medoids clustering and (**B**) hierarchical clustering in R program version 4.1.2 (The R Foundation for Statistical Computing, Vienna, Austria; https://svn.r-project.org/R-packages/trunk/cluster, accessed on 17 March 2021), (**C**) bar plot, (**D**) pie chart.

**Figure 4:**
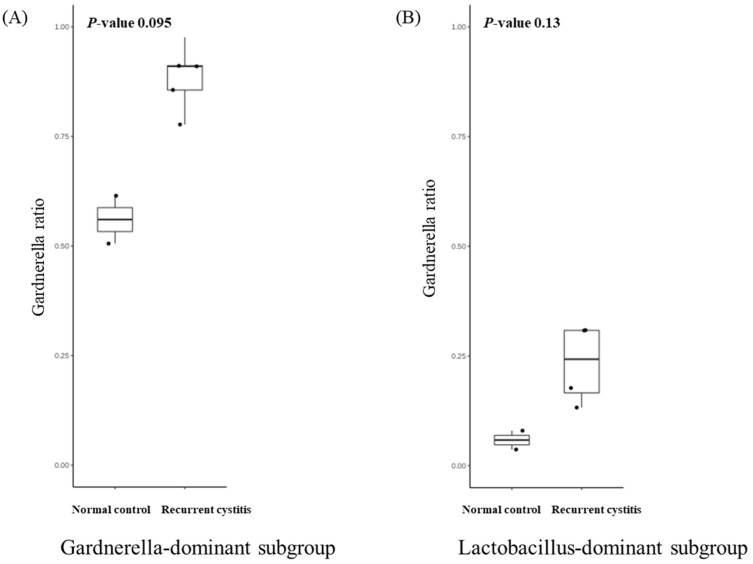
Relative abundance of *Gardnerella* in (**A**) *Gardnerella*-dominant subgroup and (**B**) *Lactobacillus*-dominant subgroup.

We investigated the presence of any patterns in the *Gardnerella*-positive group regardless of NC or rUTI status. The distribution of *Gardnerella* (+) urinary microbiota was analyzed with K-medoids clustering (Figure 3A), hierarchical clustering (Figure 3B), and a bar plot (Figure 3C), and was classified into three groups as follows (Figure 3D): Group 1 *Escherichia* urotype (*Gardnerella* is very few and *Escherichia* is dominant), Group 2 *Gardnerella* urotype (*Gardnerella* accounted for more than 50%), and Group 3 *Lactobacillus* urotype (*Gardnerella* is present in some cases, but *Lactobacillus* is predominant in more than 50%). All of Group 1 (*Escherichia* urotype) was associated with rUTI (5 rUTI, 0 NC). In Group 2 (*Gardnerella* urotype: 5 rUTI, 2 NC) or Group 3 (*Lactobacillus* urotype: 4 rUTI, 2 NC), both rUTI and asymptomatic NC were present (Figure 3A). However, in Group 2 and Group 3, the proportion of *Gardnerella* was higher in the rUTI group than the NC group, although not significant (Group 2, *p* = 0.095; Group 3, *p* = 0.13) (Figure 4).

## 4. Discussion

In this study, we found that there was no significant difference in urine microbiota results between the *Gardnerella*-positive NC group and *Gardnerella*-positive rUTI group. The *Gardnerella*-positive group could be divided into three urotypes: (1) *Escherichia*-dominant group, (2) *Gardnerella*-dominant group, and (3) *Lactobacillus*-dominant group. All *Escherichia*-dominant groups were associated with rUTI. In the *Gardnerella*-dominant and *Lactobacillus*-dominant groups, the NC and rUTI groups were mixed. In particular, bacterial vaginosis-associated strains such as *Atopobium*, *Megasphaera*, and *Ureaplasma* were detected only in the rUTI group.

Our research group has completed two papers related to the urine microbiome. The core of the first paper was that *E. coli* was the most causative strain in acute and recurrent cystitis, but the base from which *E. coli* grew, i.e., the bladder condition (commensal or pathogenic organism), was completely different [14]. In other words, the first paper was a study on the *E. coli*-dominant urotype. In this paper, the second study, we found the possibility that *Gardnerella* in the bladder could influence the uncertain pathophysiology of the dominant urotype of recurrent urinary tract infection.

A recent UTI guideline is based on antibiotic treatment based on urine culture for rUTI. In particular, continuous low-dose antimicrobial prophylaxis is recommended if rUTI persists after behavioral interventions have failed. In reality, 75% of the patients in clinical practice with rUTI are taking empirical antibiotics without undergoing tests [27]. Consequently, antibiotic resistance has increased while rUTI prevalence has not decreased [28].

Recently, through the new bacterial technique (16s RNA sequencing, EQUC), the classical perception of the bladder microbiome is being reconsidered, and the role of various microorganisms in urinary tract infection needs to be re-established [7,14]. According to a previous classical urine culture-based study, the most common causative bacteria of uncomplicated UTI were *E. coli* (58%), mixed flora (13.4%), and *K. pneumoniae* (6.5%) [29]. However, the pattern was different in our group’s previous pilot study [14]. This study, based on NGS, additionally discovered *Sphingomonas, Staphylococcus, Streptococcus*, and *Rothia* spp., which were not found in the existing culture, especially for the rUTI group. Although the NGS test has better diagnostic yield than the existing tests, it should also be noted that there are several drawbacks of the NGS test. First of all, NGS is not yet a widely used standard test method, as with urine culture, in clinical practice, and it is mainly used for research purposes [29]. In addition, the cost of NGS is more expensive than urine culture or EQUC, and when various bacteria are identified, it is difficult to interpret the results as to which bacteria are the actual pathogens [30]. Lastly, the lack of primer universality can be a major problem compared with urine culture or EQUC [13].

With the accumulation of the knowledge regarding this microbiome, the importance of the gut–vagina–bladder axis for rUTI has been increasingly emphasized. Clinical evidence of an association between the vagina and bladder is as follows. First, bacterial vaginosis is a risk factor for UTI [31]; second, UTI decreases when hormone treatment such as vaginal estrogen is administered [32,33], and lastly, there are many patients who complain of frequent UTI after sex [34]. On a microbiological basis, it has been reported that around two thirds of the bladder microbiota overlaps with the gut microbiota, and around one third of the bladder microbiota exists only in the vagina [35].

The first finding of our study was that there was no difference in the detection rate of *Gardnerella* between the NC and rUTI groups. Similar to our result, a previous study reported that *Gardnerella* was detected in 27% of the normal population, and this ratio was not significantly different from patients with urinary symptoms [10]. *Gardnerella* is often detected even in normal asymptomatic individuals. However, in the absence of symptoms due to a host–pathogen immune response, *Gardnerella* does not cause disease. Therefore, not only the detection rate of *Gardnerella* but also symptoms are important for interpretation. Taken together, *Gardnerella* can influence the uncertain pathophysiology of the dominant urotype in rUTI under the assumption that *Gardnerella* causes symptoms. In our study, in the normal group, *E. coli* did not appear even if *Gardnerella* was detected, but in the rUTI group, 22.57% of patients with *Gardnerella* were detected (Table 3). Although the clinical significance cannot be confirmed due to the small number of subjects, it shows abnormal diversity in the rUTI group. This means that bladder dysbiosis may present a differential immune response to bacterial colonization and different symptoms. In particular, *Atopobium, Megasphaera*, and *Ureaplasma*, known to be associated with bacterial vaginosis, were detected only in the rUTI group.

Second, our study suggested three patterns of *Gardnerella*-positive patients. First, in the *E. coli*-dominant group, this is the first study of humans showing that *Gardnerella* might be a covert pathogen that activates *E. coli*. It has already been shown that the vagina acts as a reservoir for uropathogens such as *E. coli*. Moreover, even short exposure of the bladder to *Gardnerella* caused bladder cell damage such as urothelial exfoliation and urothelial apoptosis in an animal study [17]. The second group (*Gardnerella*-dominant group) provides clues that increased amounts of *Gardnerella* itself may be associated with rUTI. Although rare, it has been reported that *Gardnerella* acts as a causative agent of UTIs [31]. Considering the low culture-positive rate of *Gardnerella*, the clinical significance is likely to be higher in practice. The clues from the study that the increased amount of *Gardnerella* in the *Gardnerella* urotype in the bladder may be related to rUTI are as follows. First, in these patients, no rUTI-causing bacteria were detected other than *Gardnerella*, and secondly, in this case, other vaginitis-causing bacteria were also accompanied. Taken together, we interpreted that dysbiosis caused by vaginitis strains, especially *Gardnerella*, induced symptomatic rUTI. In the case of the third *Lactobacillus*-dominant group, since the protectivity effect of *Lactobacillus* is different depending on the type of *Lactobacillus* strain, cystitis can occur even with a small percentage of *Gardnerella* in *Lactobacillus* strains with poor protectivity. However, in this study, the *Lactobacillus* strain was not analyzed, and thus further follow-up studies are required to investigate the difference between normal and rUTI strains.

Considering that both NC and rUTI existed in Group 2 (*Gardnerella*-dominant) and Group 3 (*Lactobacillus*-dominant), we can make the following inferences. Asymptomatic *Gardnerella* infection is present in the vagina, and some are self-treated [36]. Likewise, the presence of *Gardnerella* does not necessarily cause rUTI, just as the presence of *Gardnerella* does not necessarily cause bacterial vaginosis. Although *Gardnerella* itself does not have high virulence, it is affected by other risk factors for causing symptoms. Risk factors such as host immunity, *Gardnerella* proliferation, the microbiome environment, and the residence environment seem to influence the development of the phenotype (asymptomatic or rUTI). For example, it seems that recurrent cystitis tends to occur when *Gardnerella* and other bacterial vaginosis strains are co-present, which can be interpreted as the influence of the microbiome environment. In addition, the fact that the proportion of *Gardnerella* was higher in rUTI patients (although not significant) could be evidence that the amount of *Gardnerella* proliferation had an effect on phenotype expression. Overall, our results provide clues for a new pathophysiology of rUTI. The usage of antibiotics based on traditional uropathogens in Group 2 or Group 3 has weak clinical effects and may cause side effects of antibiotic resistance to occur.

We suppose that the increased number of UTIs in menopause might be caused by decreased estrogen levels and associated physical changes. Estrogen helps the growth of *Lactobacillus* bacteria in the vagina and bladder, and the proliferation of *Lactobacillus* bacteria reduces urinary tract infections. After menopause, estrogen reduction and changes in *Lactobacillus* are thought to promote UTI. In fact, estrogen replacement hormone therapy in postmenopausal women reduced cystitis by providing a microenvironment that improved the growth of normal *Lactobacillus* in the vagina [37,38,39,40,41,42]. However, in this study, it was not possible to analyze the effect of estrogen replacement, because prescription of vaginal hormones was impossible during the study period due to the shortage of medication.

Our study has several advantages. First, we demonstrated the clinical importance of the gut–vagina–bladder axis in humans, focusing on *Gardnerella* for the first time. The gut–vagina–bladder axis has been explained to some extent through microbiological and animal experiments, but studies on humans are still lacking [8,17,35]. Second, in our study, the specimen was collected by transurethral catheterization, so the bladder microbiome was well reflected without contamination. Finally, our study suggested several types and novel mechanisms of rUTI that had not been previously elucidated.

However, our study also has several limitations. First, since this study was conducted in a retrospective manner, selection bias is inevitable, especially regarding the NC group. Moreover, we could not match the cases and controls regarding their estrogen hormonal status. Due to the limitations of the retrospective study, the number of subjects corresponding to the normal control group was relatively small. In particular, in our study, normal controls were selected from those who had undergone a health check-up, and it was rare for premenopausal women to even receive a urine NGS test during a health check-up. In Korea, if a patient has urinary symptoms, the NGS test is reimbursed by the government and the cost of the NGS is low at USD 160. On the other hand, if an asymptomatic subject undergoes an NGS test for health screening purposes, the cost of the NGS is between USD 350 and 450. Due to the burden of testing costs, most asymptomatic subjects do not frequently undergo NGS testing. For this reason, it was difficult to secure additional normal controls in this study. We are aware of these limitations and are working to increase the NGS test rate in the normal population by securing research funds. Second, our study revealed the importance of *Gardnerella* infection as a covert pathogen, but did not provide a cut-off for the required amount of infection for clinical symptoms to appear. Third, the difference between the *Lactobacillus* strains in the NC group and the rUTI group could not be suggested. This requires follow-up studies, and it is believed that it will provide clues about *Lactobacillus* prophylaxis in patients with rUTI in the future. Fourth, this study did not suggest whether treating *Gardnerella* could actually improve the clinical symptoms of rUTI. In general, antibiotics used for bacterial vaginosis and those used for rUTI are different [43]. The choice of antibiotics for UTIs can also affect the vaginal microbiome. For example, the use of beta-lactam antibiotics is less effective against vaginally colonized *E. coli*, and recurrent UTIs caused by vaginal *E. coli* easily occur with such beta-lactam antibiotics [9,44]. Furthermore, *Gardnerella* is difficult to treat due to biofilm formation [45]. Metronidazole or tobramycin, which were previously recommended as therapeutic agents for *Gardnerella*, can prevent the formation of a new biofilm, but are known to have less effect on the previously formed biofilm [45]. Therefore, further studies are needed to determine which antibiotic is most suitable for rUTI caused by *Gardnerella*. Fifth, this study was able to analyze the results up to the genus level because the partial V4 region of 16S rDNA was analyzed as a target. Further follow-up studies up to the species level will be needed.

## 5. Conclusions

In summary, if conventional uropathogens are not detected in patients with rUTI, *Gardnerella* infection should be considered. Asymptomatic urine *Gardnerella* (asymptomatic bacteriuria) does not require treatment, but symptomatic patients with *Gardnerella* found in the urine may need treatment, considering the possibility of the causative organism. Moreover, even for asymptomatic cases, treatment should be considered if *E. coli* or the causative agent of bacterial vaginosis is detected in addition to *Gardnerella.* Overall, our study suggest that dysbiosis of the bladder microbiome may show different colonization and different symptoms.

## Figures and Tables

**Table 1:** Baseline characteristics of patients.

	Total (*n* = 96)	Normal Control (*n* = 18)	Recurrent Urinary Tract Infection (*n* = 78)	*p*
Age (years)	54.5 ± 14.9	47.1 ± 11.8	56.2 ± 15.1	0.291
Female	96 (100)	18 (100)	78 (100)	0.999
Menopause	61 (63.5)	8 (44.4)	53 (67.9)	0.062
Diabetes	15 (15.6)	2 (11.1)	13 (16.7)	0.558
Urinalysis				
Urine RBC (per HPF)	0 (0–400)	0 (0–400)	0 (0–30)	0.051
Urine WBC (per HPF)	0 (0–204)	0 (0–29)	0 (0–204)	0.252
Urine bacteria (per HPF)	0 (0–3+)	0 (0–3+)	0 (0–3+)	0.720
Urine protein	0 (0–2+)	0 (0–1+)	0 (0–2+)	0.679
Urine glucose	0 (0–3+)	0 (0–3+)	0 (0–3+)	0.710

NOTE: The data are presented as the mean ± standard deviation or median (min–max) for continuous variables and *n* (%) for categorical variables. Abbreviations: RBC, red blood cell; WBC, white blood cell; HPF, high-power field.

**Table 2:** Positive detection rate of *Gardnerella* species.

	Number of Patients	*Gardnerella*-Positive Samples	*p*-Value
Normal control	18	4 (22.2%)	0.677
Recurrent UTI	78	14 (18.0%)	
Total	96	18 (18.8%)	

**Table 3:** Contribution of most abundant urinary bacterial genera to *Gardnerella* positive group.

Genera	Percent Contribution in G (+) Normal Control Group			Percent Contribution in G (+) Recurrent UTI Group		
	Mean	Min	Max	Mean	Min	Max
*Lactobacillus*	55.67	8.19	96.00	24.87	0.00	86.38
*Gardnerella*	30.94	3.70	61.49	42.18	1.13	99.09
*Haemophilus*	7.58	0.00	30.33	6.52	0.00	91.33
*Kocuria*	3.60	0.00	14.38	0.01	0.00	0.13
*Tumebacillus*	1.91	0.00	7.65	0.00	0.00	0.00
*Escherichia*	0.23	0.00	0.60	22.57	0.00	96.93
*Ureaplasma*	0.06	0.00	0.12	0.32	0.00	2.56
*Atopobium*	0.00	0.00	0.00	1.74	0.00	12.73
*Pseudoxanthomonas*	0.00	0.00	0.00	0.40	0.00	5.46
*Megasphaera*	0.00	0.00	0.00	0.35	0.00	4.96
*Ruminococcus*	0.00	0.00	0.00	0.29	0	4.08
*Clostridium IV*	0.00	0.00	0.00	0.25	0.00	3.48
*Blautia*	0.00	0.00	0.00	0.33	0.00	3.20
*Alloscardovia*	0.00	0.00	0.00	0.15	0.00	2.03

Abbreviations: G (+), *Gardnerella*-positive.

## Data Availability

Not applicable.
